# Indirect plant-mediated interactions between heterospecific parasitoids that develop in different caterpillar species

**DOI:** 10.1007/s00442-023-05465-z

**Published:** 2023-10-27

**Authors:** Maximilien A. C. Cuny, Romain Pierron, Rieta Gols, Erik H. Poelman

**Affiliations:** 1 Laboratory of Entomology, Wageningen University, P.O. Box 16, 6700 AA Wageningen, The Netherlands; 2 Laboratoire Vigne Biotechnologies et Environnement, Université de Haute-Alsace, Colmar, France

**Keywords:** Tritrophic interactions, Induced plant response, Parasitoid performance, Parasitoid-mediated interactions, Indirect interaction network

## Abstract

Parasitoids induce physiological changes in their herbivorous hosts that affect how plants respond to herbivory. The signature of parasitoids on induced plant responses to feeding by parasitized herbivores indirectly impacts insect communities interacting with the plant. The effect may extend to parasitoids and cause indirect interaction between parasitoids that develop inside different herbivore hosts sharing the food plant. However, this type of interactions among parasitoid larvae has received very little attention. In this study, we investigated sequential and simultaneous plant-mediated interactions among two host–parasitoid systems feeding on *Brassica oleracea* plants: *Mamestra brassicae* parasitized by *Microplitis mediator* and *Pieris rapae* parasitized by *Cotesia rubecula*. We measured the mortality, development time, and weight of unparasitized herbivores and performance of parasitoids that had developed inside the two herbivore species when sharing the food plant either simultaneously or sequentially. Plant induction by parasitized or unparasitized hosts had no significant effect on the performance of the two herbivore host species. In contrast, the two parasitoid species had asymmetrical indirect plant-mediated effects on each other’s performance. *Cotesia rubecula* weight was 15% higher on plants induced by *M. mediator*-parasitized hosts, compared to control plants. In addition, *M. mediator* development time was reduced by 30% on plants induced by conspecific but not heterospecific parasitoids, compared to plants induced by its unparasitized host. Contrary to sequential feeding, parasitoids had no effect on each other’s performance when feeding simultaneously. These results reveal that indirect plant-mediated interactions among parasitoid larvae could involve any parasitoid species whose hosts share a food plant.

## Introduction

In response to herbivory or egg deposition, plants can produce primary and secondary metabolites and/or change their architecture and morphological traits (Kessler and Baldwin 2002). For instance, plants from the Brassicaceae family typically respond to herbivory by enhancing production of secondary metabolites called glucosinolates that have been shown to affect plant–insect interactions (Hopkins et al. 2009). Upon leaf herbivory, the level of glucosinolates increases and can even reach 40 times its initial level (Gols et al. 2018). Induced plants have not only an altered phenotype that may influence the inducer but also other herbivores interacting later with the same plant (Faeth 1986). Induced responses to feeding by herbivores may affect performance of sequentially feeding herbivores even when those are the fifth in a row of sequential episodes of herbivore attack (Fernández de Bobadilla et al. 2022). Even more so, induced responses to early season herbivores may last for several months in affecting community assembly (Poelman et al. 2010). These types of non-trophic interactions are termed “indirect plant-mediated interactions” (Faeth 1986). They play an important role in how plants affect the structure of associated insect communities (Utsumi et al. 2010; Poelman and Dicke 2014; Stam et al. 2014).

Indirect plant-mediated interactions are typically taking place in a multitrophic context in which predators and parasitoids play an important role (Utsumi et al. 2010). Parasitoids are generally small wasps that exhibit two main reproductive strategies: endoparasitoids, which lay their eggs inside the host, and ectoparasitoids, which lay their eggs outside the host (Godfray 1994). Both groups of parasitoids fully develop on the expense of the herbivore that is their host. Thereby, they are often influenced by the effect of induced plant responses on herbivore performance and quality. For example, parasitoids can suffer from the negative effect of plant-induced response on their host through the reduction in nutrient availability (Ode 2006), although this is more pronounced for generalists compared with specialist parasitoids (Gols et al. 2008; Bukovinszky et al. 2012). Sequestration of enhanced levels of defense compounds by specialist herbivores may kill parasitoid larvae (Kazana et al. 2007). Moreover, parasitoid eggs developing inside an herbivorous host feeding on an herbivore-induced plant suffer less from immune system encapsulation by the host hemocytes (Bukovinszky et al. 2009).

These multitrophic indirect plant-mediated interactions are characterized by specificity in the outcome of the interactions. For example, they depend on the plant species and herbivores involved. Induced plant responses are often adapted to the attacker guild and/or species (Heidel and Baldwin 2004; Mewis et al. 2006; Kessler and Halitschke 2007). Upon damage, plants perceive and recognize herbivore-associated molecules in order to fine tune their response (Felton and Tumlinson 2008; Mithöfer and Boland 2008; Bonaventure 2012). As a result, plant induction by different herbivore species may result in either positive (susceptibility) or negative (resistance) effects on the subsequent herbivore, depending on the feeding guild as well as level of host plant specialization of both herbivores involved in the plant-mediated interaction (Kaplan and Denno 2007; Mertens et al. 2021). Moreover, the density, feeding location, severity of herbivory, and time interval between feeding by the two herbivore species may affect the strength of the interaction. Plant-mediated species interactions are often stronger when a first herbivore has been feeding for several days on a plant before the second herbivore arrives than when herbivores feed simultaneously (Erb et al. 2011). This may be due to the gradual build-up of induced resistance after the onset of herbivory.

Parasitoids themselves may also modify indirect plant-mediated interactions through herbivore-induced plant responses (Kaplan et al. 2016). Parasitoids can mediate quantitative changes in herbivore damage, such as a reduction or an increase of the amount of plant tissue consumed by their host (Harvey 1996; Ode et al. 2016; Cuny and Poelman 2022). Moreover, parasitoids modify qualitative aspects of herbivory, such as the composition of the herbivore saliva (Poelman et al. 2011b; Tan et al. 2018, 2019), altering the way plants perceive the identity of their herbivorous attacker. This may, in turn, affect herbivores feeding on the same plant via indirect plant-mediated interactions (Cusumano and Volkoff 2021; Poelman and Cusumano 2022). For example, plant-phenotypic changes induced by parasitized herbivores increase the performance of unparasitized herbivores and reduce the oviposition preference of adult herbivores compared to plants induced by unparasitized caterpillars (Poelman et al. 2011b; Cusumano et al. 2018, 2021). If two parasitized herbivores feed on the same plant, parasitoid larvae developing in different hosts from the same species can affect each other through indirect plant-mediated interactions. So far, indirect plant-mediated interactions involving interactions between parasitoid larvae have only been demonstrated in one study (Poelman et al. 2011a). More research is needed to have a better understanding of indirect plant-mediated interactions among parasitoid larvae in nature.

In theory, two parasitoids that do not share the same host range are not expected to interact directly. However, the concept of indirect interactions, such as indirect plant-mediated interactions, challenges our understanding of the limitations of species interactions. Indirect plant-mediated interactions provide a mechanism that allows for the possibility of interactions between parasitoids with different host ranges, as long as their respective hosts share a common host plant (Poelman and Cusumano 2022). Additionally, indirect plant-mediated interactions among parasitoids have only been tested sequentially, and virtually nothing is known about simultaneous feeding on the same plant.

In this study, we examined indirect plant-mediated interactions between two host–parasitoid systems feeding on *Brassica oleracea* plants: *Mamestra brassicae* (Lepidoptera: Noctuidae) parasitized by *Microplitis mediator* (Hymenoptera: Braconidae) and *Pieris rapae* (Lepidoptera: Pieridae) parasitized by *Cotesia rubecula* (Hymenoptera: Braconidae). We measured the effect of plant induction by parasitized and unparasitized caterpillars on the performance of unparasitized caterpillars as well as the two parasitoid species in their respective host species. In parallel, we also tested the hypothesis that parasitoids more prominently affect performance of other parasitoids when their hosts are feeding sequentially from the food plant rather than feeding simultaneously due to time lags in establishment of induced plant phenotypes. Our results provide important insights in how indirect plant-mediated interactions could affect multitrophic interactions in natural and agricultural ecosystems.

## Materials and methods

### Plants and insects

As host plants, we used wild cabbage *Brassica oleracea* which grows naturally along the coastline of England. Seeds were collected from the Kimmeridge population in Dorset, UK (Gols et al. 2008). This plant has been shown to respond differently to parasitized and unparasitized caterpillar hosts (Zhu et al. 2015). Plants were germinated and seedlings were transferred to 2-L pots containing peat soil (Lentse potgrond No. 4; Lentse Potgrond BV, Lent, The Netherlands). Pots were placed in a greenhouse, providing the plants with a 16:8 (light: dark) photoperiod with SON-T light (tubular sodium lamps, moles of quanta; 500 μmol·m-2·s-1) (Philips, Eindhoven, The Netherlands) in addition to daylight, at 18–26 °C and 40–70% relative humidity. When the plants were four weeks old, they were fertilized weekly with 100 mL of nutrient solution (Kristalon, Nutritech System, Moscow, Russia, concentration 3 g/L, [16N:6P:20 K:3 Mg]).

Two lepidopteran herbivore species were used in our experiments: *Pieris rapae* and *Mamestra brassicae*. *Pieris rapae* is a specialist feeding on Brassicaceae that is able to deal with high levels of glucosinolates (i.e., plant secondary metabolites) (Wittstock et al. 2004), while *M. brassicae* is considered a generalist and is less adapted to this type of chemical defense (Gols et al. 2008). We used two solitary endoparasitoids from the Braconidae family that do not share the same host and are both considered specialists. *Cotesia rubecula* parasitizes *P. rapae* (Brodeur et al. 1998), while *Microplitis mediator* parasitizes *M. brassicae* (Malcicka and Harvey 2014). All insects were routinely cultured on *Brassica oleracea* Cyrus under greenhouse conditions (18–26 °C and 40–70% relative humidity).

### Experimental approach

**Fig. 1:**
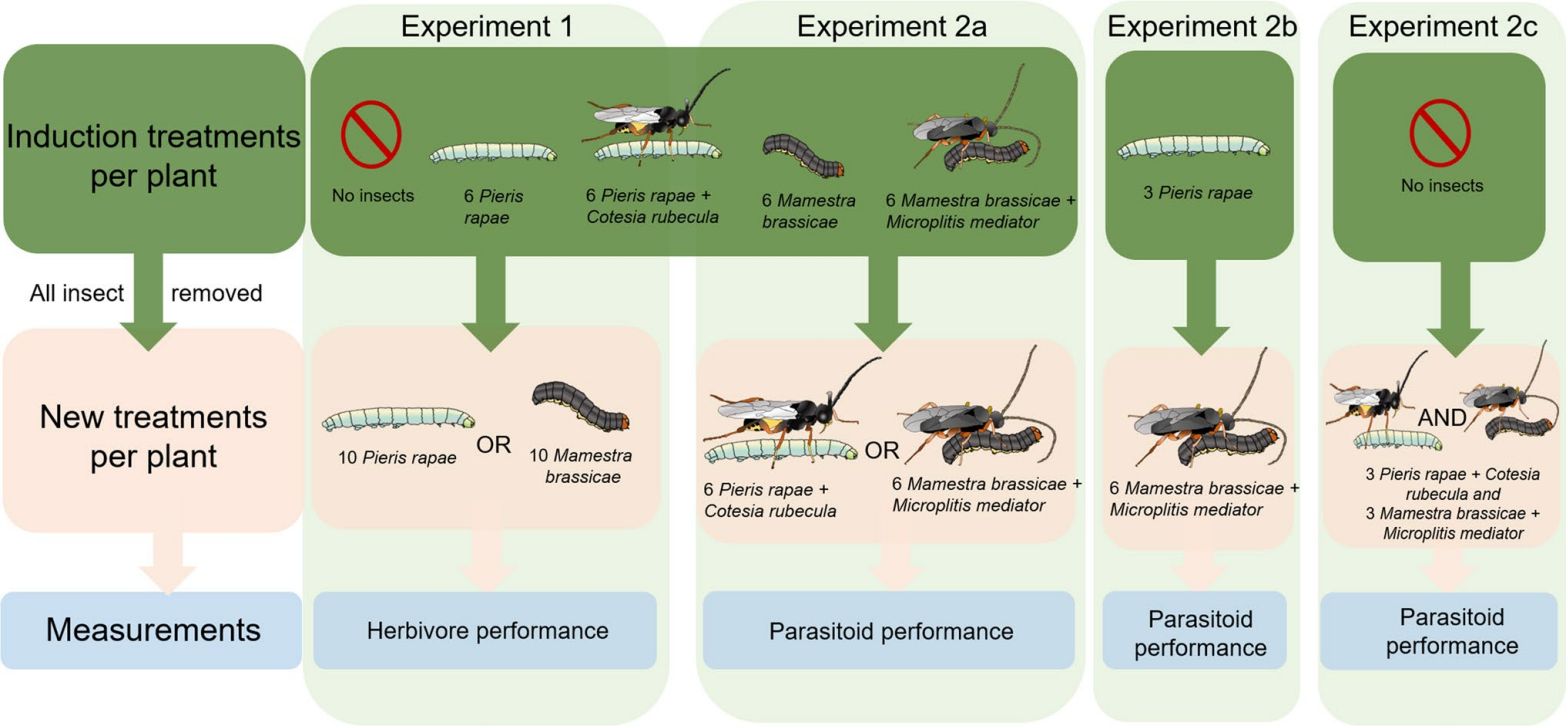
Experimental design to investigate plant-mediated interactions among parasitoid larvae of *Cotesia rubecula* and *Microplitis mediator* that develop in two different herbivore species, *Pieris rapae* and *Mamestra brassicae,* respectively. *Brassica oleracea* plants were left unchallenged (no insects) or were induced by unparasitized or parasitized herbivores. In experiment 1, the induced plants were used to identify how caterpillars and parasitoids developing in an herbivore affect performance of new unparasitized caterpillars that feed sequentially on the plant. In experiment 2, the same 5 treatments of induced plants were offered to parasitized herbivores to identify how parasitoids developing in different herbivores affect each other (2**a**). In addition, we added two treatments: (2**b**) only three *Pieris rapae* on the induced plants, to test the effect of herbivore density when compared to the six *P. rapae* in experiment 2**a**; (2**c**) no insect inducing the plant and then parasitized caterpillars from the two systems to investigate simultaneous feeding

To unravel whether parasitoids that develop in one herbivore host species may affect parasitoids developing in a different one when feeding on the same food plant, we conducted two experiments (Fig. 1). In all the experiments, we induced plants with first instar, one-day-old caterpillars. In the first experiment we focused on how each parasitoid species developing in its respective host caterpillar affects development of subsequently feeding unparasitized caterpillars of both host species. In the second experiment, we tested whether parasitoids developing in their respective host species affect conspecific or heterospecific parasitoids developing in a second herbivore. In parallel, we added two treatments to the second experiment: (i) parasitized herbivores feeding simultaneously on the plants to test the effect of the induction timing and (ii) a treatment where plants were induced with half the number of unparasitized caterpillars to test the density effect. Together the results of these experiments reveal whether parasitoids affect each other via performance effects on each other’s host or that these effects are more intricate without affecting host herbivore performance traits.

#### Experiment 1: plant-mediated effects on unparasitized herbivores

Six-week-old *B. oleracea* plants were individually covered with a net and infested with a first round of herbivory according to one of the following treatments (sixteen plants per treatment): 1) no herbivory, 2) six unparasitized *P. rapae*, 3) six *P. rapae* parasitized by *C. rubecula*, 4) six unparasitized *M. brassicae*, or 5) six *M. brassicae* parasitized by *M. mediator*. Neonate caterpillars of each species were individually parasitized (Poelman et al. 2014) by their corresponding parasitoid one day prior to plant infestation. After nine days when the parasitoid larvae were full grown and nearly all of them had egressed from their caterpillars for pupation, we removed all herbivores from all the plant treatments. The following day, we infested half of the plants from each treatment (eight plants) with ten unparasitized *P. rapae*, and the other eight plants with ten unparasitized *M. brassicae*. This second round of herbivory was used to measure host performance. *P. rapae* were allowed to develop on the plant until pupation, while *M. brassicae,* which pupate in the soil, were transferred into boxes with one cm of soil when they reached the wandering stage in search of a pupation site. Performance was assessed by i) fresh weight of the pupae, ii) adult emergence time from introduction of the caterpillars onto the plant, and iii) mortality rate, i.e., number of caterpillars not developing into adults relative to the number that were initially introduced. Pupae of both herbivores were stored in plastic tubes at 22 °C to measure development time until adult emergence.

#### Experiment 2a: plant-mediated effects on parasitoids

Six-week-old *B. oleracea* plants were infested with the same five main treatments as mentioned above, with thirty plants per treatment that were each covered by a net to prevent herbivores from moving to neighboring plants. After nine days when nearly all parasitoids had egressed from their caterpillars, we removed all herbivores from all the plant treatments. The day after removing all the insects from the plants, half of the plants from the five main treatments received six *P. rapae* parasitized by *C. rubecula*, while the other half received six *M. brassicae* parasitized by *M. mediator* (fifteen plants per treatment). We used only six caterpillars per treatment (compared to ten unparasitized caterpillars in the first experiment), because parasitized caterpillars are harder to obtain. Parasitoid cocoons were collected and individually placed in plastic tubes and checked for emergence five times per day. To quantify parasitoid performance, we measured i) difference in parasitoid development time (in hours, starting from the first adult parasitoid emergence) and ii) adult dry weight. The sex of the adult parasitoids was determined under a stereomicroscope. Before measuring parasitoid dry mass on a microbalance, the parasitoids were dried for 2 days in an oven at 80 ^∘^C.

#### Experiment 2b: effects of herbivore density

In addition to the five treatments described above, fifteen other plants were infested with only three unparasitized *P. rapae* during the first round of herbivory (instead of 6). After pupation, the caterpillars were removed and the plants received six *M. brassicae* parasitized by *M. mediator*. This treatment was used to compare how the amount of herbivory, i.e., three or six *P. rapae* feeding during the first round of herbivory, affects plant-mediated effects on parasitoids. The performance of parasitoids was measured as described above.

#### Experiment 2c: effects on timing on plant-mediated interactions between parasitoids

Thirty additional undamaged plants were left without insects during the first round of herbivory and received simultaneously three parasitized *P. rapae* and three parasitized *M. brassicae*. This treatment was used to compare how time interval, i.e., simultaneous feeding or sequential feeding by parasitized caterpillars, affects plant-mediated interactions among parasitoids that develop in different host herbivores on the same food plant. The performance of parasitoids was measured as described above.

### Statistical analyses

In all our models, we used plant induction treatment as a fixed factor and plant individual as a random factor. We used linear mixed models to analyze the development time of herbivores and parasitoids, except for unparasitized *M. brassicae* in the first experiment. Because of the non-normal distribution of the residuals of these data, we used a generalized linear mixed model with a Poisson distribution. The mortality of unparasitized caterpillars and parasitoids was analyzed using generalized linear mixed models with a binomial distribution, while we analyzed their weight with linear mixed models. We analyzed the dry weight of males and females jointly for *Cotesia rubecula* because of a low female ratio. Females and males from both species were pooled for developmental time and mortality. When a significant difference was found with a mixed model, we used Tukey’s post hoc tests for pairwise comparisons between all the treatments. All statistical analyses were performed with R (version 4.0.4, R Core Team 2021), using the lme4 packages for mixed models.

## Results

### Experiment 1: plant-mediated effects on unparasitized herbivores

**Fig. 2:**
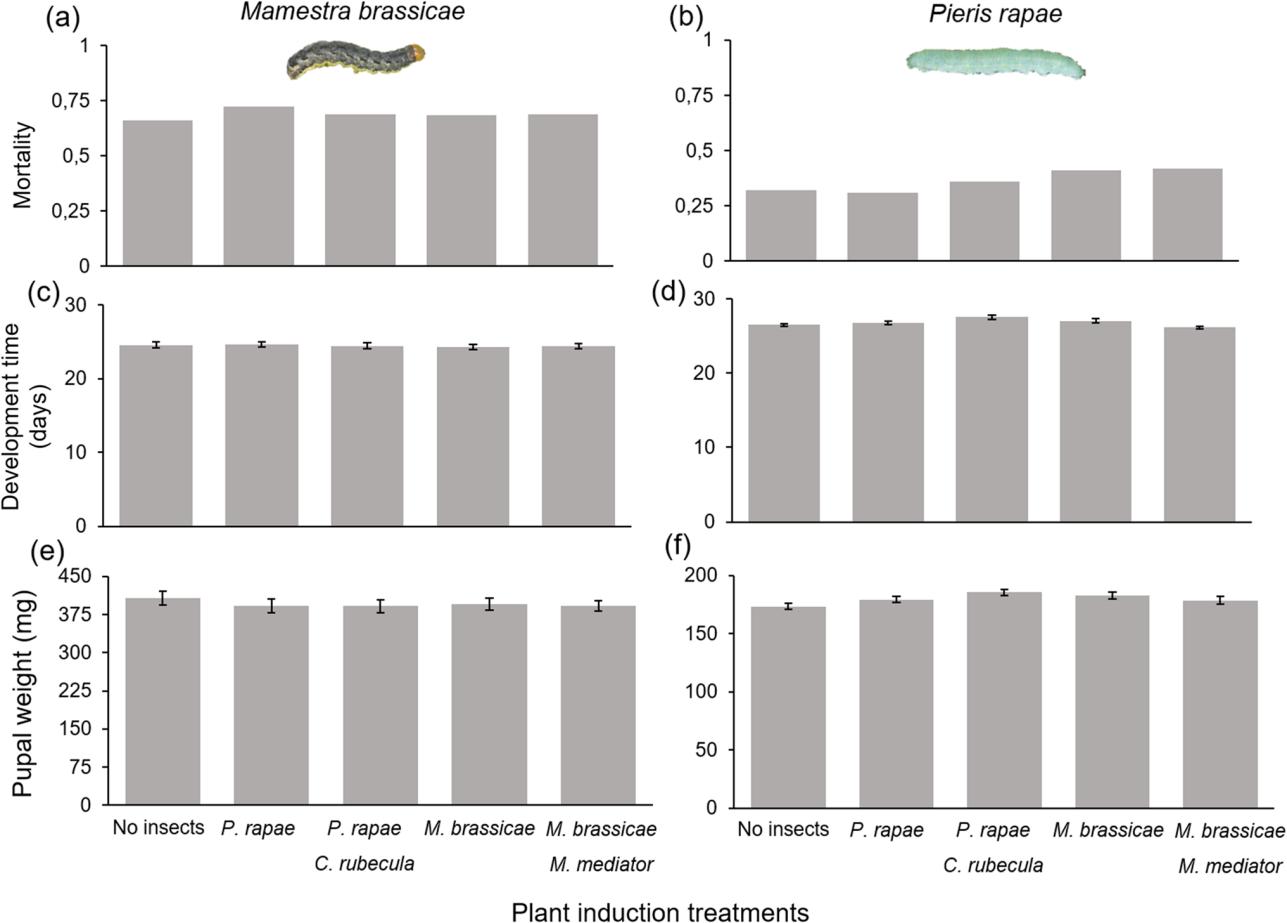
Performance parameters of *Mamestra brassicae* (**a**, **c**, **e**) and *Pieris rapae* (**b**, **d**, **f**) developing on *Brassica oleracea* plants that received different induction treatments. Bars are means (± SEM)

Feeding by a first generation of parasitized or unparasitized *P. rapae* and *M. brassicae* caterpillars had no plant-mediated effects on the development time (χ^2^_(4)_ = 7.8, *P* = 0.097), mortality (χ^2^_(4)_ = 2.12, *P* = 0.71) and pupal weight (χ^2^_(4)_ = 4.94,* P* = 0.29) of unparasitized *P. rapae* that were subsequently feeding from the induced plants. Similar results were found for the development time (χ^2^_(4)_ = 0.06, *P* = 0.99), mortality (χ^2^_(4)_ = 1.52, *P* = 0.82), and pupal weight (χ^2^_(4)_ = 1.04, *P* = 0.90) of unparasitized *M. brassicae* (Fig. 2).

### Experiment 2a: plant-mediated effects on parasitoids

**Fig. 3:**
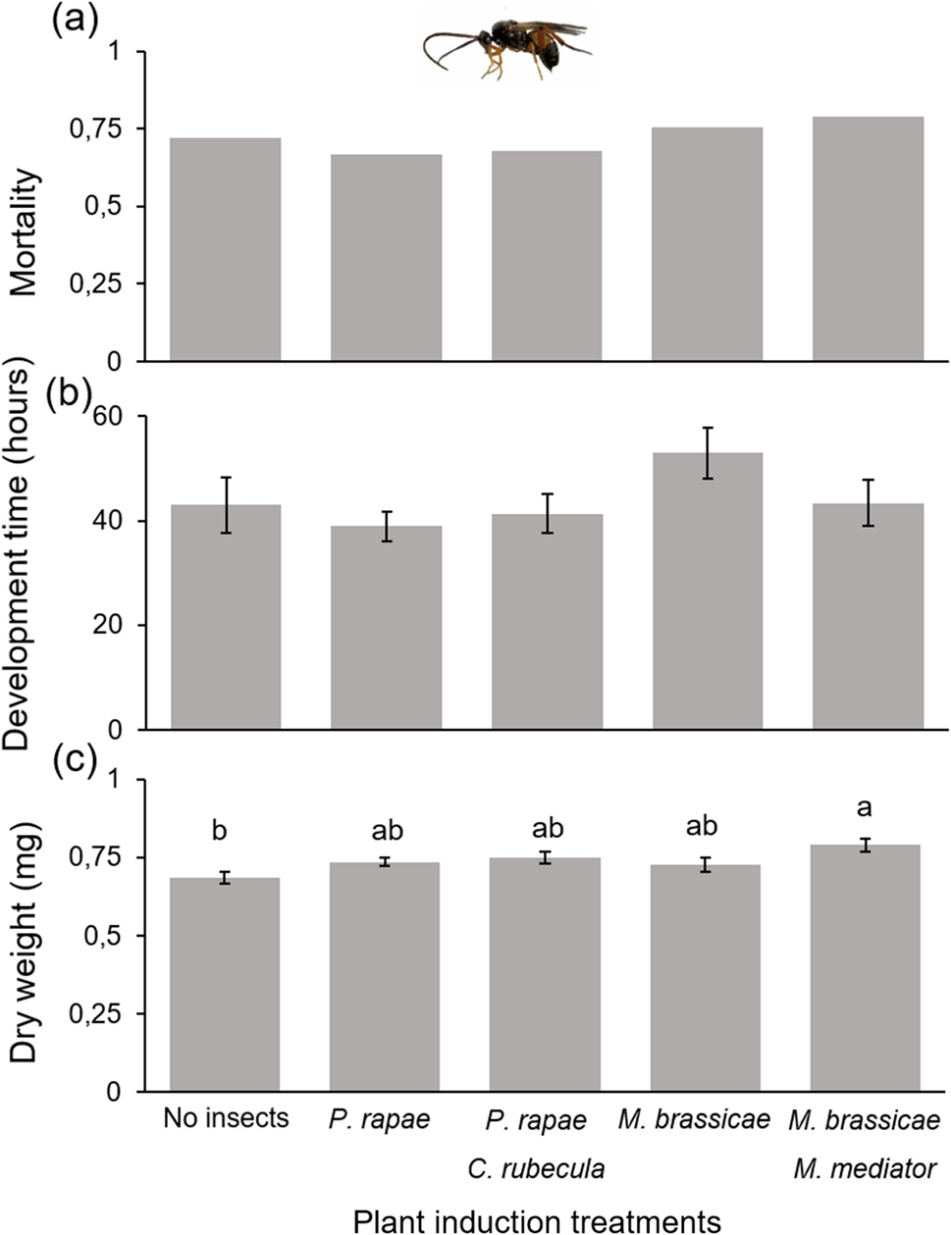
Performance parameters of *Cotesia rubecula* parasitoids whose host fed on plants previously induced by different treatments of parasitized and unparasitized caterpillars. **a** mortality ratio of *C. rubecula,* **b** development time measured in hours from oviposition until adult emergence, and **c** *C. rubecula* adult dry weight. Bars are means (± SEM). Different letters indicate significant differences

**Fig. 4:**
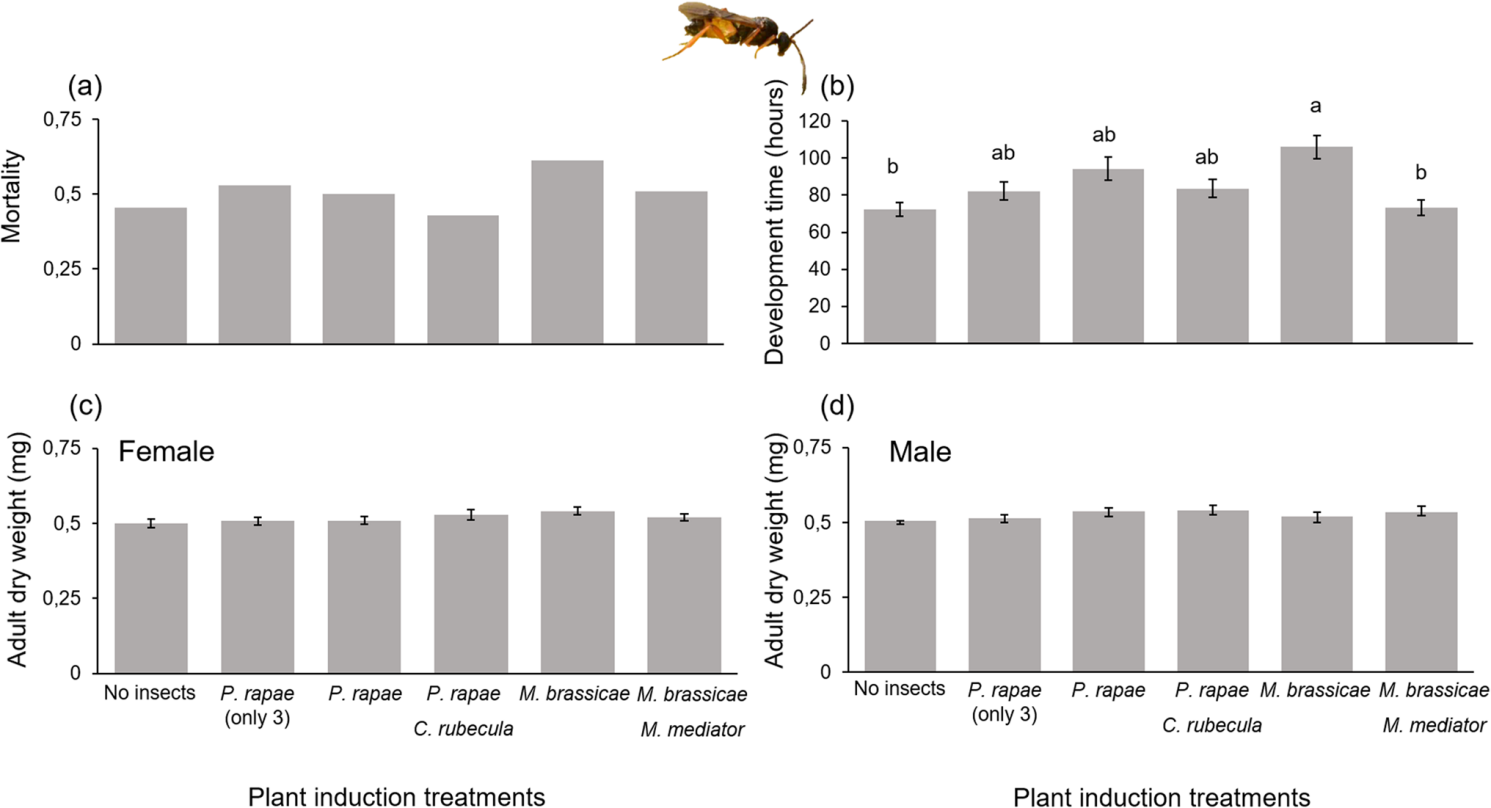
Performance parameters of *Microplitis mediator* parasitoids whose host fed on plants previously induced by different treatments of parasitized and unparasitized caterpillars, including a treatment with only three *P. rapae* (instead of six). **a** mortality ratio of *M. mediator,* **b** development time measured in hours from oviposition until adult emergence and **c** female *M. mediator* adult dry weight, and **d** male *M. mediator* adult dry weight. Bars are means (± SEM). Different letters indicate significant differences

Mortality (χ^2^_(4)_ = 4.72, *P* = 0.32) and development time (χ^2^_(4)_ = 3.82, *P* = 0.43) of *C. rubecula* were not affected by induction treatment. However, the dry weight of adult *C. rubecula* was significantly higher when their hosts were feeding on a plant that had been induced by *M. brassicae* larvae parasitized by *Microplitis mediator* compared to control plants (χ^2^_(4)_ = 14.22, *P* = 0.007) (Fig. 3). Dry weights of *C. rubecula* on the other three induction treatments were intermediate, but did not differ from those on the no-herbivory and *M. brassicae–M. mediator * induction treatment (Fig. 3). Plant induction treatments had no significant effect on the mortality (χ^2^_(5)_ = 6.89, *P* = 0.23) and dry weight of *M. mediator* adult parasitoids, both for females (χ^2^_(5)_ = 5.65, *P* = 0.34) and males (χ^2^_(5)_ = 4.68, *P* = 0.46). Development time of *M. mediator* was significantly shorter when their hosts were feeding on control plants and plants induced by *M. brassicae* caterpillars parasitized by conspecific parasitoids compared to plants induced by unparasitized *M. brassicae* (χ^2^_(5)_ = 20.94, *P* < 0.001) (Fig. 4). The heterospecific parasitoid *C. rubecula* and its host *P. rapae* did not significantly affect performance of *M. mediator* developing in *M. brassicae*.

### Experiment 2b: herbivore density does not affect plant-mediated interactions between parasitoids

The lower density of unparasitized *P. rapae* caterpillars (three) was not statistically different from the higher density (six) and had no significant effect on the development time, mortality, and adult female and male dry weight of *M. mediator* parasitoids developing in *M. brassicae* (Fig. 4).

### Experiment 2c: feeding timing affects plant-mediated interactions between parasitoids

**Fig. 5:**
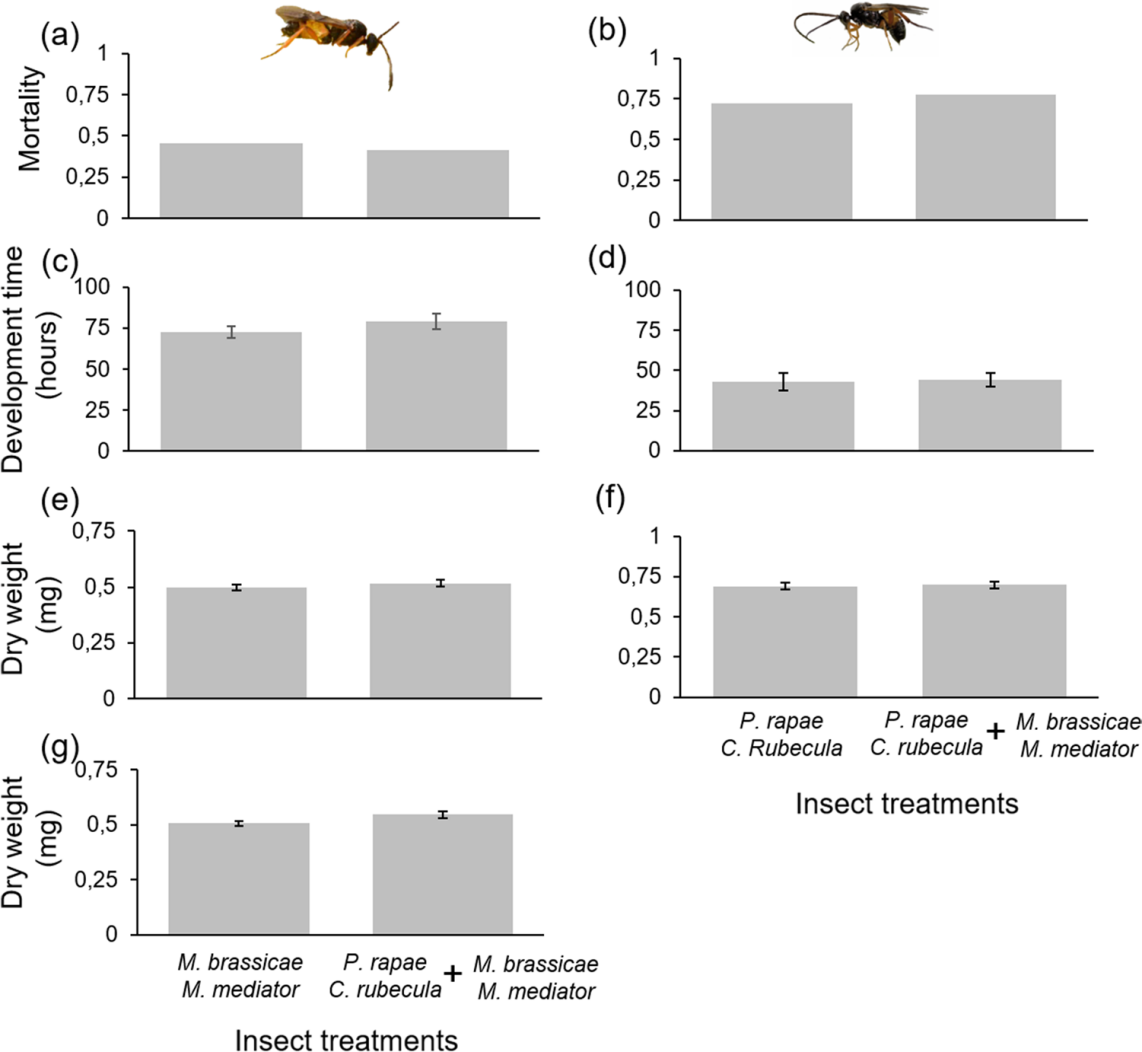
Performance parameters of *Microplitis mediator* (left) and *Cotesia rubecula* (right) parasitoids whose hosts fed on untreated plants, either alone or simultaneously with parasitized caterpillars from the other system. **a** mortality ratio of *M. mediator,* **b** mortality ratio of *C. rubecula*, **c** development time measured in hours from the first *M. mediator* adult emergence, **d** development time measured in hours from the first *C. rubecula* adult emergence, **e** *M. mediator* male adult dry weight, **f** *C. rubecula* adult dry weight (both males and females), and **g** *M. mediator* female adult dry weight. Bars are means (± SEM)

Contrary to sequential feeding, parasitized *P. rapae* and *M. brassicae* caterpillars feeding simultaneously on the same food plant that was not previously induced did not affect each other’s performance (Fig. 5). We found no significant effect of simultaneous feeding on the parasitoids mortality (*M. mediator*: χ^2^_(1)_ = 0.34, *P* = 0.56; *C. rubecula*: χ^2^_(1)_ = 0.62, *P* = 0.43), development time (*M. mediator*: χ^2^_(1)_ = 0.98, *P* = 0.32; *C. rubecula*: χ^2^_(1)_ = 0.06, *P* = 0.8), and adult dry weight (*M. mediator* males: χ^2^_(1)_ = 1.51, *P* = 0.22; *M. mediator* females: χ^2^_(1)_ = 0.85, *P* = 0.36 *C. rubecula*: χ^2^_(1)_ = 0.25, *P* = 0.61) compared to feeding alone.

## Discussion

In this study, we deepened understanding of indirect plant-mediated interactions between parasitoid larvae developing in separate hosts. We show that these understudied interactions are not limited to parasitoids whose host range overlap (Poelman et al. 2011a), but may occur between parasitoids associated with different herbivore species. Previous damage by parasitized and unparasitized caterpillars on *B. oleracea* plants did not affect performance of unparasitized *P. rapae* and *M. brassicae* subsequently feeding on the same plant. Contrary to their host, when the subsequent herbivores feeding on the induced plants were parasitized, we found that induction with *M. brassicae* parasitized by *M. mediator* affected both parasitoid species positively. Dry weight of *C. rubecula* was significantly increased when feeding on plants previously induced by *M. mediator*-parasitized herbivores, compared to undamaged plants. On the other hand, *M. mediator* parasitoids developed significantly faster when their hosts were feeding on plants induced by conspecific parasitized hosts, with no effect of plant induction by *C. rubecula*. However, no plant-mediated effects were observed on the performance of parasitoids when parasitized caterpillars fed simultaneously on the same food plant.

### Interactions do not affect unparasitized herbivores

Parasitized caterpillars from the two systems had no apparent effect via indirect plant-mediated interactions on subsequent unparasitized caterpillars. These results are not surprising for *Pieris rapae*, which is a specialist of Brassicaceous species, well adapted to their defensive compounds, such as glucosinolates (Wittstock et al. 2004). However, these results are more surprising for *Mamestra brassicae* which is considered as a generalist, less adapted to glucosinolates (Gols et al. 2008), although a previous study showed that it may be as well adapted to glucosinolates as *Plutella xylostella*, a specialist herbivore (Poelman et al. 2008). Although the level of specialization may play a role in our results, it is important to note that we only tested two parasitoid species. Therefore, more research is needed to unravel the specific impact of parasitoid specialization on their plant-mediated effects. In a similar study, Poelman et al. (2011a) also found no effect of previous induction by unparasitized and *C. rubecula*-parasitized *P. rapae* on the performance of subsequent unparasitized *P. rapae*. However, *P. rapae* developmental time was significantly increased when feeding on plants induced by *C. glomerata*-parasitized larvae, compared to undamaged plants. In another study (Cusumano et al. 2021), plants induced by parasitized caterpillars increased the performance of unparasitized ones, but the experimental design differs from ours as unparasitized caterpillars were fed with cut leaves induced by mechanical damage and saliva and the relative growth rate of caterpillars was measured after 48 h.

### Interactions are asymmetrical and facilitating

We observed an asymmetrical, facilitating (with a positive effect) indirect plant-mediated interaction between two parasitoid species with different hosts. Plant induction by *M. mediator*-parasitized herbivores positively affected the dry weight of *C. rubecula* parasitoids. On the contrary, *M. mediator* parasitoids were not affected by plants induced by *C. rubecula*-parasitized herbivores. In a similar study, an asymmetrical sequential indirect plant-mediated interaction was found between *Cotesia glomerata* and *C. rubecula* parasitoids developing in *P. rapae* larvae on *B. oleracea* plants (Poelman et al. 2011a). Yet, in this case, *C. rubecula* had an antagonistic effect on the survival of *C. glomerata* and no effects were found on performance of *C. rubecula*. The asymmetrical antagonistic indirect plant-mediated interactions between two parasitoid species with overlapping host ranges could be adaptive in order to limit competition for hosts when the parasitoid emerge (Poelman et al. 2011a). Asymmetrical indirect plant-mediated interactions could also be affected by the level of adaptation of the herbivorous host to the food plant. For example, specialists may detoxify toxic compounds, which could reduce any negative effect of plant induction on the parasitoid. We also found a facilitating indirect plant-mediated interaction between conspecific parasitoids. *Microplitis mediator* significantly reduced the development time of subsequent conspecific parasitoids developing on the same plant. Such facilitation could be the result of an adaptive extended phenotype of the parasitoids that alter plant response via their host (Cusumano et al. 2018; Zhu et al. 2018) in order to increase performance of their host and as a result of this, their own fitness (Tan et al. 2018; Cusumano et al. 2021). Alternatively, parasitoids are under a strong selective pressure to modify their host physiology, which optimizes parasitoid larval development. Therefore, the observed indirect plant-mediated interactions among parasitoid larvae could only be by-products of physiological changes upon parasitism, without evolutionary pressure, resulting in unpredictable outcomes of indirect plant-mediated interactions among parasitized herbivores (Cuny et al. 2022). The fact that plants react differently to parasitized herbivores can be hypothesized to be an adaptive strategy, reducing the resources invested in its defensive response when the herbivore is anyway going to die soon from parasitism (Tan et al. 2020). Alternatively, being attacked by parasitized herbivores may be detrimental, as they may not be able to recognize the identity of the attacker and to respond with the adapted strategy. From an evolutionary point of view, this could be a case where the interests of plants and parasitoids differ, which may result in a more diffuse selection on plant defensive response against herbivores (Cuny and Poelman 2022).

### Underlying mechanisms

During this study, we did not measure any plant traits in response to herbivory. While this does not invalidate our results, we do not know the underlying mechanisms of parasitoid plant-mediated interactions. However, we can hypothesize that parasitism affected the oral secretion composition of their hosts (Poelman et al. 2011b; Cusumano et al. 2018), which in turn affects the plant’s ability to recognize the herbivore species, inducing a different plant defensive response. Because such alteration can affect the plant in unpredictable ways, we can speculate that (i) parasitized *Mamestra brassicae* induced a particular response to the plant, (ii) this response alters the physiology of the parasitized herbivore (immune system, nutritive value, toxic plant compounds ingested), and (iii) this physiological change is beneficial for parasitoids, without having a significant effect when the herbivore is not parasitized.

### Interactions are not affected by herbivore density

The lower number of *P. rapae* caterpillars feeding on the plant did not affect the performance of subsequent *M. mediator* parasitoids developing in *M. brassicae*. This result suggests that the effects of indirect plant-mediated interactions among parasitoids are caused by qualitative changes in the herbivorous host and food plant, and not by quantitative effects on herbivore growth or by variation in amount of leaf damage by herbivores. However, it has to be noted that we only tested two caterpillar densities and that we did not measure leaf damage. In addition, negative effects caused by quantitative changes could eventually arise with more caterpillars and more damage.

### Interactions are especially sequential, and only affect parasitoids

Our results revealed that parasitized caterpillars simultaneously feeding on the same plant did not result in indirect plant-mediated interactions between parasitoids. This was particularly clear for the dry weight of *C. rubecula* that was significantly increased by the previous feeding of *M. mediator*-parasitized caterpillars, but not when feeding simultaneously. In response to insect feeding, *B. oleracea* plants slowly increase their levels of glucosinolates during up to two weeks (Gols et al. 2018). Therefore, simultaneous feeding of caterpillars on the same plant may be too short to observe an effect on their performance. Alternatively, simultaneous feeding by two herbivores may lead to a more general response that is less efficient than an adapted response against one specific herbivore species (Fernández de Bobadilla et al. 2022). No other study tested the effects of simultaneous feeding of parasitized herbivores.

### Implications

Parasitoids are ubiquitous in many natural and agricultural ecosystems in which they play an important role in structuring trophic interactions. Despite the very low number of studies investigating parasitoid plant-mediated interactions, we can speculate that such interaction could also happen in other systems with non-lepidopteran herbivores. For instance, the aphid *Myzus persicae* parasitized by *Aphidius colemani* induced a different plant response compared with unparasitized aphids (Vaello et al. 2018). In our study, we contribute to the awareness that different parasitoid species are involved in complex indirect non-trophic interactions mediated by plants. We fine-tuned the theory about this phenomenon by showing that (1) indirect plant-mediated interactions between parasitoids may lead to facilitation in addition to the known antagonistic outcome (Poelman et al. 2011a); (2) simultaneous feeding of parasitized caterpillars on the same plant may be too short for plant induction to have an apparent effect on their performance, as it is the case in sequential feeding; (3) contrary to parasitoids, their herbivorous hosts were not affected by indirect plant-mediated interactions; and (4) qualitative rather than quantitative changes in herbivory induced by parasitoids seem to be responsible for the effects observed in indirect plant-mediated interactions among parasitoids.

Our work identifies that even parasitoids that were thought to never interact because of their different host ranges, can be involved in indirect plant-mediated interactions as long as their respective herbivorous hosts share a common food plant. As a result, indirect plant-mediated interactions among parasitoid larvae may be even more widespread than previously thought and should be recognized as an important indirect interaction type in food webs.

## Data Availability

The data that support the findings of this study are available from the corresponding author upon reasonable request.
